# An ecological momentary music intervention for the reduction of acute stress in daily life: A mixed methods feasibility study

**DOI:** 10.3389/fpsyg.2022.927705

**Published:** 2022-09-29

**Authors:** Anja C. Feneberg, Urs M. Nater

**Affiliations:** ^1^Department for Clinical and Health Psychology, Faculty of Psychology, University of Vienna, Vienna, Austria; ^2^University Research Platform “The Stress of Life (SOLE) – Processes and Mechanisms underlying Everyday Life Stress”, University of Vienna, Vienna, Austria

**Keywords:** ambulatory assessment, autonomic nervous system, ecological momentary intervention, feasibility, HPA axis, mobile health, music, stress management

## Abstract

**Background:** Despite the growing potential of mobile-based technologies, innovative interventions targeting the reduction of acute stress in daily life remain under-researched. Music listening is an easy-to-administer activity that is associated with lower levels of biological and self-reported stress. However, the application of music as an intervention in moments of acute stress in daily life remains to be examined. We developed a just-in-time intervention delivering music in moments of stressful experiences in daily life and tested its feasibility using a mixed methods approach.

**Methods:** In this uncontrolled pilot study, the ecological momentary music intervention (EMMI) was tested by 10 chronically stressed women aged 23.5 ± 3.3 years. Over 18 consecutive days, whenever participants reported stressful experiences, they were encouraged to listen to a self-compiled playlist. Subjective stress levels and saliva samples were assessed at three time points per stress report (T_0_, upon reporting a stressful situation; T_1_, directly after music listening/15 min after T_0_ in case of no music listening; T_2_, 15 min after T_1_). We analyzed app-based log data, in-the-moment responses, questionnaire data, and semi-structured interview data.

**Results:** On average, participants’ compliance with the study protocol lay at 70%. Overall, 65 stressful experiences were reported, 51 of which were followed by music listening, for an average duration of 12:53 min. Complete data (i.e., self-reports and saliva samples at all three time points) were provided for 46 stressful experiences. Participants reported immediate relaxation and distraction through music listening. The interviews revealed that the intervention was easy to use and that music listening in moments of perceived stress was viewed as a new and pleasant activity. Several aspects of the protocol (e.g., number of items and prompts) were identified, which should be improved in future studies.

**Conclusion:** Since repeated stressful experiences in daily life can pose a threat to physical and mental integrity, interventions that are easily applicable and deliver support when needed most are necessary. Following minor adaptations, the EMMI can be considered as a feasible approach to target psychobiological stress responses in daily life, which is worthy of investigation in future larger-scale trials.

## Introduction

Stress is a well-known experience in many people’s daily life and an omnipresent phenomenon of society as a whole. While stress responses are adaptive in the short term, recurrent and long-term stress can increase the risk for the development and manifestation of mental and somatic disorders (McEwen, 1998). To reduce the individual and societal burden of stress and stress-related disorders, easy-to-administer and cost-effective early intervention and prevention strategies are of utmost importance. Music listening can be seen as a powerful tool in this regard given its capacity to reduce both subjective and biological stress levels and its easy accessibility in daily life (Linnemann et al., 2015; Witte et al., 2019). Furthermore, due to digital technologies, interventions can be transferred from clinical settings to people’s day-to-day lives, potentially revolutionizing modern health care systems (Schueller et al., 2017). However, only seldomly has research attempted to directly intervene in experiences of acute stress in everyday life (i.e., “just-in-time” interventions) (Smyth and Heron, 2016). Moreover, the effectiveness of music listening as a treatment for immediate stress reduction in daily life settings remains to be examined. To close these research gaps, we developed an ecological momentary music intervention (EMMI) and tested its feasibility in the natural environment of chronically stressed individuals.

According to the transactional stress model, “stress” is experienced when an individual faces a situation that is appraised as important and challenging and is perceived as exceeding one’s coping resources (Lazarus and Folkman, 1984). The human stress response involves psychological changes (e.g., in cognition and affect) as well as increased activity of the body’s two main stress response systems: the hypothalamic–pituitary–adrenal (HPA) axis and the autonomic nervous system (ANS). Both systems interact with other circuits in the body, both centrally and in the periphery, resulting in focused attention and increased energy availability as well as suppressed acute immune and reproductive functioning, among other things (Stratakis and Chrousos, 1995). These psychobiological adaptations prepare the individual mentally and physically to cope with the stressful situation, underlining the highly functional nature of the acute stress response. However, extreme, persistent, and/or frequently recurring stress reactions might foster the dysregulation of the stress response and associated biological systems, thereby contributing to negative health outcomes in the long term (Stratakis and Chrousos, 1995; McEwen, 1998).

Importantly, besides major life events (e.g., divorce, death of a significant other, and natural disasters), which do not typically occur on a day-to-day basis, there is a growing research interest in ever-present minor stressors of daily life (e.g., arguments, extra work, and traffic jams). For instance, in an interview-based daily diary study, Almeida and colleagues (Almeida et al., 2002) reported that minor stressors occurred on around 40% of study days and were related to higher levels of physical symptoms and negative mood. In addition to daily diary studies, ecological momentary assessment (EMA) methods are being increasingly used to investigate time-varying psychobiological phenomena and their dynamic associations with daily stressors in the natural environment, that is with high ecological validity and (minimal) retrospective bias (Shiffman et al., 2008). A recent EMA study documented that both having experienced a minor stressor and anticipating a stressor in the next few hours were related to higher subsequent negative affect (Neubauer et al., 2018). Furthermore, the current literature indicates that the way individuals respond to minor stressors in daily life (e.g., with increased/prolonged negative affect), rather than the occurrence of stressors *per se*, is associated with elevated inflammation (Sin et al., 2015) and is predictive of mental (Charles et al., 2013) and somatic (Leger et al., 2018) health impairment up to 10 years later. Such findings emphasize that to mitigate negative health consequences, the reduction of stress responses in everyday life represents a promising target for interventions.

Despite the accumulated knowledge concerning the significance of daily stressors and the stress-health relationship, as well as the technological advancements accomplished over the past decades, evidence-based interventions that support individuals to regulate their stress levels in daily life (so-called “ecological momentary/ambulatory assessment interventions”) are still scarce (Loo Gee et al., 2016). While there are numerous commercially and freely available “stress management” apps, the vast majority of these have not been scientifically evaluated and many include non-evidence-based or even potentially harmful contents (Coulon et al., 2016; Lau et al., 2020). In addition, recent advances have been made in the development of digital web-based interventions (mostly implemented via internet sites). Centered on cognitive behavioral principles and relaxation techniques, participants in these interventions are supposed to (self-) acquire stress management skills over several sessions (Hintz et al., 2015; Ebert et al., 2016). As with more traditional face-to-face stress management programs, most digital interventions are not specifically designed to deliver support in moments when individuals are experiencing acute stress. Instead, the transfer of knowledge and generalizations to a variety of different situations are left to the individual. A just-in-time intervention approach, by contrast, is based on the idea that an intervention is most effective when it is delivered contingently upon the momentary needs of an individual in a given context (Heron and Smyth, 2010). Although this approach seems particularly promising for the purpose of downregulating acute stress responses, there is still a relative lack of agreed-upon guidelines on how to best capture (i.e., measure), and intervene in response to, acute stressful experiences occurring in everyday life (Epel et al., 2018; Zawadzki et al., 2019). Consequently, innovative interventions targeting person- and context-varying experiences require intense piloting with regard to feasibility aspects (e.g., participant compliance, usage rates, treatment adherence, user satisfaction) in order to gather the necessary information to plan subsequent larger-scale trials (O’Cathain et al., 2019).

Music listening is an extremely popular leisure activity across different age groups and cultures (International Federation of the Phonographic Industry, 2019). It also encompasses several advantages as an intervention tool, such as its low cost, easy accessibility, and lack of detrimental side effects. Several meta-analyses and reviews have summarized the beneficial effects of music interventions on psychological, neuroendocrine, and autonomic stress parameters (Pelletier, 2004; Thoma and Nater, 2011; Chanda and Levitin, 2013; Witte et al., 2019). However, only recently, research has moved from lab and clinical contexts into individuals’ daily life with preliminary, albeit promising evidence. For instance, a series of EMA studies from our own research group indicated that merely listening to music in daily life can be effective in downregulating psychological and biological stress markers in healthy and clinical populations, which might, in turn, translate into beneficial health outcomes such as reduced somatic complaints (Linnemann et al., 2015, 2018; Feneberg et al., 2021; Wuttke-Linnemann et al., 2019). Besides these observational studies, a small number of intervention studies have aimed to examine immediate music-induced effects on psychological and biological stress levels in daily life contexts (Carissoli et al., 2015; Helsing et al., 2016; Kappert et al., 2019; Neal-Barnett et al., 2019; Giordano et al., 2020; Kirk and Axelsen, 2020). However, most of these were not tailored to specifically target acute stressful experiences, involved a rather inflexible design disregarding situational aspects, failed to consider subjective and biological stress markers in tandem, and yielded mixed results.

Consequently, there is a relative lack of just-in-time interventions targeting acute experiences that are appraised as stressful, especially when considering music listening as a treatment. Therefore, we developed the EMMI based on a larger framework of existing stress theories, the transactional stress theory (Lazarus and Folkman, 1984) and the allostatic load model (McEwen, 1998). The EMMI is centered on the individual’s appraisal of a situation as stressful (Lazarus and Folkman, 1984), while improved recovery from stressful experiences is assumed to prevent detrimental health-related consequences (McEwen, 1998). Furthermore, previous empirical work on the beneficial effects of music listening on psychobiological stress levels and health outcomes outlined above guided the development of the EMMI. We considered it necessary to thoroughly investigate the feasibility of the EMMI in a first step.

We sought to investigate feasibility as indicated by (a) general compliance with the study protocol, (b) number and nature of reported stressful experiences in daily life, (c) compliance with saliva sampling and saliva quantity in moments of self-reported stressful experiences, (d) treatment (non-)adherence, that is frequency, characteristics, and duration of (non-)music listening episodes in moments of stressful experiences, (e) perceived immediate effects of music listening, and (f) participants’ perspectives on the usability of and satisfaction with the EMMI as well as study participation overall. To investigate these aspects, we used a combination of app-based log data, EMA and non-EMA questionnaires, and semi-structured interview data.

## Materials and methods

### Study design

**Figure 1:**
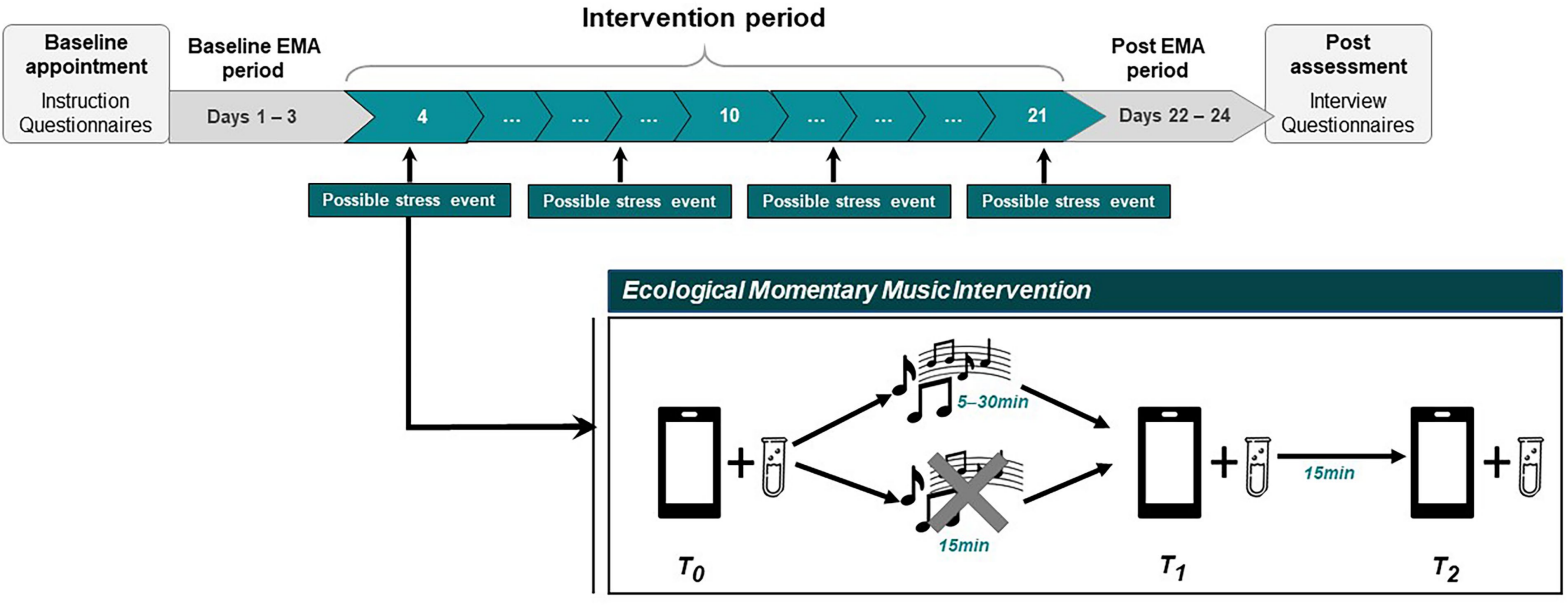
Schematic overview of the course of the study. During the 18-day intervention period, participants are prompted six times per day to report whether they feel stressed at the moment (“yes”/”no”) and are instructed to self-initiate a data entry each time they feel stressed. Upon reporting a momentary stress experience (T_0_), participants are encouraged to listen to their study playlist, which can be complied with or rejected. Post-(non-)music listening assessments (T_1_, T_2_) are included to capture immediate and time-delayed effects on subjective stress levels and biological stress markers (salivary cortisol, salivary alpha-amylase). EMA, ecological momentary assessment. Tube icon provided by https://www.flaticon.com.

In this uncontrolled pilot study, chronically stressed women aged 18–35 years used the EMMI in their everyday life. An overview of the study design is displayed in Figure 1. Data were collected before the intervention period (= baseline) and after the intervention period (= post) using online (non-EMA) questionnaires as well as EMA methods on three successive days, respectively, in order to determine psychological and biological stress profiles. During the 18-day intervention period, whenever participants reported an acute stressful experience, they were instructed to listen to a previously self-compiled music playlist (see below) for between 5 and 30 min, with assessments of psychological and biological stress levels taken before (= T_0_) and directly after (= T_1_) music listening. As HPA axis responses to external (negatively and positively valenced) stimuli are observed with a time delay (Schlotz et al., 2008), we additionally included a second post-music listening assessment 15 min after T_1_ (= T_2_). Participants could also choose not to listen to music (e.g., in stressful situations that did not permit music listening or when they did not want to listen to music) and instead continue with their current activity. In this case, assessments of psychobiological stress levels were scheduled upon reporting a stressful situation (= T_0_), 15 min later (= T_1_), and again 15 min afterward (= T_2_).

The first participant was enrolled in November 2019. Due to the outbreak of the COVID-19 pandemic in Austria in March 2020, the study was paused for around 6 months and recruitment resumed in October 2020. Before continuing testing, we decided to adapt the study procedures such that face-to-face contact between research staff and participants was reduced to a necessary minimum (see procedure for further details).

### Participants

We recruited women who lived in or close to Vienna, Austria. Previous research suggests sex/gender-specific differences regarding psychobiological stress responses (Strahler and Nater, 2017) and the psychobiological effects of music listening (Wuttke-Linnemann et al., 2019). Thus, we decided to recruit exclusively (self-identified) women in the present pilot study. In addition, we aimed at recruiting individuals who perceived feelings of stress in their daily life for a longer period of time, thereby increasing the chance that participants reported a sufficient number of events that allowed them to make use of the intervention. Therefore, interested women were included if they exceeded the sex-specific mean score of chronic stress on the Perceived Stress Scale (10-item version, PSS-10) based on norm values from a representative German sample (i.e., > 13) (Klein et al., 2016). In addition, we applied the following inclusion criteria in order to rule out potential confounding effects on psychobiological stress markers (Strahler et al., 2017): age between 18 and 35 years; body mass index (BMI) between 18.5 and 30 kg/m^2^; no current mental or somatic disorders; no current medication or engagement in lifestyle behaviors that affect the HPA axis or ANS functioning; and no individuals working in music-related studies/professions. A full overview of the inclusion criteria is shown in Table 1.

**Table 1:** Inclusion criteria.

General	PSS-10 score > 13
	Age between 18 and 35 years
	Body mass index between 18.5 and 30 kg/m^2^
	Self-identifying as male or female
	Fluency in speaking German
	No shift work
Mental and somatic disorders	No acute depressive episode and no depressive disorder with less than 5 years of remission
	No alcohol abuse in the past 6 months
	No substance abuse or substance-related addictions
	No eating disorders in the past 5 years
	No lifetime bipolar disorder, psychotic disorder, borderline personality disorder
	No other current mental disorders
	No chronic somatic disorders
	No intake of psychotropic drugs
Music-related aspects	No perfect pitch
	No music-related studies (i.e., university-level education) or profession
	No impairment of hearing capability
Lifestyle aspects	Smoking ≤7 cigarettes per week
	No drug consumption (except for alcohol/cannabis) during the past year
	No cannabis consumption in the past 2 weeks
	No regular practice of relaxation techniques (yoga, meditation, progressive muscle relaxation, autogenic training)
Menstrual cycle/Pregnancy	No pregnancy/current breastfeeding
	Regular menstrual cycle
	No hormonal contraception
	No premenstrual syndrome

Information on inclusion criteria was based on participant self-report unless otherwise indicated. Cut-off scores for the PSS-10 (German 10-item version of the Perceived Stress Scale, Klein et al., 2016) refer to sex- and age-specific mean norm values of a representative German sample.

All participants provided written informed consent and were able to discontinue study participation at any time without negative consequences. The protocol was approved by the Ethics Review Board of the University of Vienna and all procedures were conducted in accordance with the Declaration of Helsinki in its latest version.

### Ecological momentary music intervention

The present study involves a just-in-time intervention that delivers participant-selected music in moments of perceived stressful experiences in daily life. Both self-selected and researcher-selected music have been shown to be related to lower stress levels (Witte et al., 2019). However, self-selected music is thought to have a more profound effect on health-related outcomes, possibly due to a higher liking of and memories associated with the music together with increased feelings of control (Labbé et al., 2007; Lunde et al., 2019). Furthermore, self-selected music is a more ecologically valid stimulus, as it represents typical music listening behaviors of everyday life (Eerola and Vuoskoski, 2013). Therefore, in the present study, we instructed participants to bring a self-selected playlist including 10–25 songs to a first laboratory appointment, which they would listen to during the intervention period [see (Helsing et al., 2016) for a comparable procedure]. Participants were advised to choose songs that they liked, perceived as relaxing/calming, and would be willing to listen to repeatedly during the intervention period. The music could be from any genre with or without lyrics. If participants had access to a personal on-demand music streaming service without auditory advertisements, they could create their study playlist directly within their personal accounts, which were then used throughout the study. In any other case, participants were asked to bring their songs in mp3 format on a portable device (e.g., USB stick), from which they were transferred to the (study) smartphone at the appointment and played via a music app on the smartphone (Musik Player–MP3 Player, InShot Inc.).

During the intervention period, each time a participant reported experiencing a situation appraised as stressful (according to a prespecified definition, see section “Procedure”), the study app encouraged her to listen to her study playlist on the smartphone. To enable the possibility to optimally adjust the treatment (i.e., music listening) to their momentary situation, participants should choose a listening duration between 5 and 30 min using a timer within the app, although we recommended a music listening duration of 20 min based on previous observational findings (Linnemann et al., 2018). Participants were free to listen to the music through head−/earphones or loudspeakers and could choose any of the songs from their playlist. In addition, participants had the option to not listen to music after reporting a stressful experience, for instance if their current situation did not permit music listening or if they did not wish to listen to music. Participants were advised to listen to their study playlist exclusively when instructed by the app to do so, but to otherwise refrain from listening to the playlist in order to rule out any potentially confounding effects. Besides this restriction, participants could continue with their regular music consumption behavior and listen to music as desired.

### Procedure

An overview of the study procedure is displayed in Figure 1. Recruitment was undertaken via social media outlets and word of mouth. The study advertisement specifically targeted women who subjectively felt stressed for a longer period of time. Interested individuals who had contacted the study team by email underwent a telephone-based screening interview in order to check the inclusion and exclusion criteria (see Table 1). Upon inclusion, participants were invited to our laboratory at the University of Vienna, Austria, where they received detailed study instructions and completed online questionnaires via Unipark (Questback GmbH). Furthermore, they were instructed on the handling of the study app for the collection of self-reported data (movisensXS, Movisens GmbH) and on the collection of saliva samples using the passive drool method with prelabeled polypropylene tubes (SaliCap®, IBL International GmbH) for the later analysis of biological stress markers (salivary cortisol, salivary alpha-amylase; data not reported). Participants could use their own smartphones or were provided with a study smartphone in the case of incompatibility with the study app. To ensure a representative sampling of daily life experiences, participants were encouraged not to change their daily routines and other music habits while taking part in the study. At the end of the appointment, participants were given a study manual with further in-depth information and contact details in case of questions. On the evening of the first day of the baseline EMA period, participants were telephoned by a member of the study team to inquire about any technical difficulties or problems.

For the entire data collection period, participants were required to initiate a data entry every time they experienced a situation appraised as stressful (i.e., event-contingent data entries). This was defined as “experiencing a situation that is personally important and unpleasant and that seems hardly or not at all manageable through one’s own efforts at the moment,” based on the transactional stress theory proposed by Lazarus and Folkman (1984). Examples of how this definition corresponds to daily stressors in participants’ daily life were discussed during the baseline appointment and the definition could be accessed throughout the whole study period within the app. While acknowledging that “feeling stressed” is an individual and situationally varying perception, this procedure aimed to promote a theoretically justified, mutual understanding of “stress” and thus standardized reporting among participants (Harkness and Monroe, 2016).

To control for potential differences in psychobiological stress levels between weekdays and weekends (Skoluda et al., 2016), EMA data collection started on the next Monday, Tuesday, or Wednesday following the baseline appointment. The following 24 days were split into three periods: a 3-day baseline period, an 18-day intervention period, and a 3-day post period. We opted for a duration that we assumed would allow us to observe several acute stressful experiences while keeping the burden of study participation as low as possible. Assuming that on around 40% of days, participants would report at least one stressful experience (Almeida et al., 2002), each participant could be expected to report at least one stressful experience on around 7 days for a 18-day long trial, which we deemed suitable for the feasibility study. Furthermore, an intervention period of 18 days results in the fact that the exact same weekdays would fall into the baseline- and post-period, respectively, so these periods would be easier to compare. On the final day of the baseline and intervention period, respectively, participants were sent a brief message through the study app informing them that a new study period would start the next day and reminding them of the changes in the sampling protocol. In each assessment period, we used a combination of event-contingent and signal-contingent assessments.

Assessment schedules were exactly the same for both the baseline and the post period: Participants should first self-initiate a data entry directly upon awakening, which was followed by a prompt 30 min later. In addition, a semi-random schedule was chosen, in which participants were prompted by the app to answer several questions concerning momentary feelings and behaviors at five measurement time points distributed between 10 am and 9 pm, with a minimum of 30 min between two successive prompts. Answering could be delayed for a maximum of 60 min. A final data entry should be self-initiated before going to bed. At each data entry before 7 pm, participants were also asked to provide a saliva sample. In addition, participants were asked to self-initiate a data entry each time they perceived a stressful situation (according to the previously provided definition) and to provide a saliva sample.

The assessment schedule for the intervention period included six semi-random data entries distributed between 8 am and 8 pm, with at least 45 min between two successive prompts and a maximum delay time of 60 min. These prompts only included one single question, asking whether participants felt stressed at the moment of the prompt (yes/no). In addition, participants should self-initiate a data entry each time they perceived an acute stressful situation by tapping a button displayed within the app. In either case, several questions regarding characteristics of the current situation and momentary stress levels appeared (= T_0_). After responding, participants were encouraged to listen to their study playlist, which they could accept or reject. If they accepted, participants then needed to select a music listening duration between 5 and 30 min and to switch to the music app containing their playlist. Once the selected duration expired, participants were prompted by the app to stop listening to music and to answer several questions concerning their momentary stress levels and music-related aspects (= T_1_). If participants decided not to listen to music, an automatic prompt appeared 15 min after the initial data entry (= T_1_). Fifteen minutes after T_1_, participants were again prompted by the app to answer several short questions. Alarms at T_1_ and T_2_ could not be postponed. At each of these three measurement time points (T_0_, T_1_, T_2_), participants were instructed to provide a saliva sample for the analysis of salivary biomarkers. In addition, a self-initiated data entry before going to bed was required on every day of the intervention period, for which participants received a reminder message at 9 pm.

After the 24 days of data collection, participants were invited to a final laboratory appointment, in which they handed over the saliva samples and study smartphone (if applicable). Moreover, participants completed several online questionnaires and underwent a semi-structured interview.

Due to the COVID-19 pandemic, Austria imposed far-reaching lockdown measures starting in mid-March 2020, which temporarily prevented the further testing of participants. In autumn 2020, the rules were eased and research with humans could be restarted following strict hygiene rules. We resumed data collection in October 2020 and adapted the study protocol slightly in order to comply with the necessary guidelines. In-person contact between participants and research staff was reduced to a necessary minimum, and involved a short meeting at the laboratory at the beginning and end of the study to hand over the study materials and obtain written informed consent. The delivery of study instructions and the post-monitoring interview (PMI) took place via video calls using Zoom (Zoom Video Communications Inc.).

Upon completion of study participation, participants were reimbursed with 120€, irrespective of the number of data entries and reported stressful experiences. In the case of premature termination of the study, participants received partial reimbursement.

### Measures

#### Psychometric measurements

The German 10-item version of the Perceived Stress Scale (PSS) was administered during the telephone interview as a measure of chronic stress (Klein et al., 2016). The scale is widely used to measure “the degree to which situations in one’s life are appraised as stressful” (p. 387; Cohen et al., 1983), including experiences of unpredictability, uncontrollability, and overload, during the past month. The German translation provides good internal consistency and construct validity, showing associations with depression, fatigue, procrastination, and reduced life satisfaction. Scores range from 0 to 40, with higher scores indicating higher chronic stress. For inclusion in the study, participants needed to exceed a score of 13, which is the mean score for women aged 20–39 years according to norm values from a representative German sample (Klein et al., 2016). Internal consistency in the present study was acceptable (Cronbach’s alpha 0.74).

The Beck Depression Inventory-II (BDI-II) was applied at the beginning of the study in order to determine depressive symptom severity in the past two weeks (Hautzinger et al., 2006). The BDI-II is a widely used 21-item questionnaire with satisfactory internal consistency and test–retest reliability. Sum scores range from 0 to 63, with scores from 0–13 indicating no or minimal depressive symptoms, 14–19 mild, 20–28 moderate, and > 29 severe depressive symptoms.

#### General compliance

General compliance with the study protocol was determined based on the accepted number of scheduled data entries using the log data of the study app. This includes data entries for scheduled prompts and self-initiated data entries upon awakening (baseline, post period) and at bedtime (baseline, intervention, post period), but does not include self-reports of stressful experiences. In addition, we report the response latency (i.e., time elapsed from the auditory signal to starting the response) and the response duration (i.e., time elapsed from starting to finishing the response).

#### Usage, compliance with, and perceived effects of the EMMI

To evaluate usage, compliance, and perceived effects of the EMMI, we assessed the number and nature of reported stressful experiences, compliance with saliva sampling and saliva quantity, number and duration of (non-)music-listening episodes, and self-reported immediate effects of music listening during the intervention period.

##### Reported stressful experiences

Participants were instructed to report every stressful situation that they experienced during study participation. Reporting could be realized either by pressing a button displayed in the study app throughout the whole study period, or by responding to one of six alarms that were scheduled each day during the intervention period (see section “Procedure”). When participants reported a stressful experience, they were asked several questions, including how long they had been feeling stressed, and to indicate the nature of the stressor by choosing from a list of stressors adapted from the Daily Inventory of Stressful Events (Almeida et al., 2002; see Supplementary Table S1, for the list of items). In addition, participants answered items regarding their current stress level, mood, fatigue, and biobehavioral covariates (data not reported). Moreover, on each evening before going to bed, participants were asked to report whether they had experienced stressful situations during the day, and if so to report the number of events and whether they had tracked these directly during the day. In case stressful events were not recorded during the day, participants were asked to briefly describe the situation(s) and their experiences (see Supplementary Table S1).

##### Saliva sampling compliance and quantity

Participants accumulated saliva in the oral cavity for 2 min, tracked by a timer within the app, and subsequently transferred the saliva into polypropylene tubes using a straw (SaliCap®, IBL International GmbH). Each tube was prelabeled with a unique code that participants had to enter into the app to enable us to check sampling compliance and to assure a correct assignment of subjective and biological data (Schlotz, 2019). Participants were instructed to store the collected saliva samples in their refrigerator/freezer compartment at home until the final laboratory visit. Then the samples were kept frozen at −20°C until shipment to the Biochemical Laboratory, University of Vienna. As stress perception has been shown to modulate salivary flow rate in humans (Gholami et al., 2017), we determined whether participants collected enough saliva in moments of acute stressful experiences. For this purpose, we weighed each SaliCap tube before and after sampling, as weights ≤0.2 g indicate that the specimen might be insufficient to analyze both cortisol and alpha-amylase.

##### (Non-)music listening episodes

After reporting a stressful experience, participants could enter in the app whether or not they wished to listen to music. If participants chose not to listen to music, they were asked to briefly describe, through an open-format item, what circumstances prevented them from doing so. If participants chose to listen to music, they selected their preferred duration, switched to their playlist, and listened to music until an alarm indicated that the chosen duration had passed and they should stop (=T_1_). Participants then answered several questions regarding the music listening situation and perceived music characteristics (see Supplementary Table S1). We determined the frequency and characteristics of (non-)music listening episodes based on self-reports at T_0_ and T_1_. In addition, response durations for T_0_, T_1_, and T_2_ (i.e., scheduled 15 min after T_1_) and response latencies for T_1_ and T_2_ data entries were calculated as an indicator of compliance in moments of acute stressful experiences.

Music listening was also tracked objectively through an additional app installed on the smartphone (Pano Scrobbler, kawaiiDango), which tracked music listening behavior and transferred the data to the online platform Last.fm (Last.fm Ltd.), from which music listening data (title, artist, date, time of starting a track in hh:mm) could be downloaded. The app recorded all songs that were listened to for at least 50% of their individual duration. These objective music listening data allowed us to control for treatment adherence and to gather data on objective music characteristics (e.g., genre, tempo).

##### Perceived immediate effects

With respect to perceived immediate effects of music listening in moments of stressful experiences, at T_1_, participants reported the extent to which the music had helped them to “relax,” “be distracted,” “calm down,” “create a nice atmosphere,” “be revived,” “evoke strong feelings,” and “express negative feelings,” on Likert scales ranging from 1 (“not at all”) to 5 (“very much”). These items are based on the Brief Music in Mood Regulation questionnaire (Saarikallio, 2012).

#### Usability and participant satisfaction

In an online questionnaire, participants were asked to rate the usability of and their satisfaction with the EMMI at the end of their study participation. Since there was no available comprehensive self-report scale assessing usability and satisfaction deemed suitable for the present context, we selected several items from existing scales and adapted them slightly. Overall, we included nine items from the System Usability Scale (Brooke, 1996), three items from the Usability Questionnaire (Lund, 2001), and four items proposed by Loo Gee and colleagues (Loo Gee et al., 2018) targeting user satisfaction. These 16 items (see Table 2) were all rated on a Likert scales ranging from 1 (“strongly disagree”) to 5 (“strongly agree”).

**Table 2:** Participant satisfaction and usability.

	(Rather) agree^a^ (rating of 4 or 5)	Partly^a^ (rating of 3)	(Rather) disagree^a^ (rating of 1 or 2)	Median	IQR
*Participant satisfaction*					
I found the intervention helpful	4 (40%)	3 (30%)	3 (30%)	3	2.25
I would apply the intervention in the future	4 (40%)	3 (30%)	3 (30%)	3	2.5
The skills I learned from the intervention help me a lot in my everyday life^b^	4 (40%)	3 (30%)	2 (20%)	3	2.5
I am satisfied with the intervention	6 (60%)	2 (20%)	2 (20%)	4	1.25
I would recommend the intervention to a friend	4 (40%)	3 (30%)	3 (30%)	3	2
The intervention was fun to use	3 (30%)	2 (20%)	5 (50%)	2.5	2.25
*Usability*					
I found the intervention unnecessarily complex	1 (10%)	3 (30%)	6 (60%)	2	2
I think the intervention was easy to use	7 (70%)	1 (10%)	2 (20%)	4	2.25
I think that I would need the support of someone with technical skills to be able to use the intervention	–	1 (10%)	9 (90%)	1	0
I found the various functions of the intervention were compatible with one another	8 (80%)	2 (20%)	–	4	0.25
I think the intervention was too unpredictable	–	–	10 (100%)	2	1
I would imagine that most people would learn to apply the intervention very quickly	8 (80%)	1 (10%)	1 (10%)	4	1.25
I found the intervention very cumbersome to use	1 (10%)	3 (30%)	6 (60%)	2	1.25
I felt very confident using the intervention	9 (90%)	1 (10%)	-	4.5	1
I needed to learn many things before I could get going with the intervention	–	–	10 (100%)	1	0.25
The intervention was easy to understand	10 (100%)	–	–	4.5	1

^a^Items were rated on a 5-point Likert scale [1 (“do not agree”), 2 (“rather do not agree”) 3 (“so so”), 4 (“rather agree”), 5 (“fully agree”)]. For the sake of interpretability, ratings were grouped into the three categories of “(Rather) agree” (ratings of 4 or 5), “Partly” (rating of 3), and “(Rather) disagree” (ratings of 1 or 2).

^b^Missing value for one participant. IQR, Interquartile range.

In addition, a self-developed, semi-structured PMI was conducted with each participant by research staff (postgraduate students with a Bachelor’s degree in psychology, supervised by AF and UN). The interview took around 20 min and was audiotaped and transcribed. The PMI was applied in order to gain a more in-depth understanding of participants’ experiences with the EMMI. Questions covered integration of the EMMI into everyday life, participants’ stressful experiences, music listening behavior, and satisfaction with study participation.

### Analytical approach

Frequency statistics of quantitative data were calculated using MS Excel (Microsoft 365) and IBM SPSS, Version 25.0 and visualized using the ggplot2 package (Wickham, 2016) in R Studio 3.6.2 (R Core Team, 2020). Transcribed interviews were analyzed qualitatively using thematic analysis (Braun and Clarke, 2006; Joffe, 2012) with the software MAXQDA (VERBI GmbH). Thematic analysis is a stepwise procedure involving (1) familiarization with the data, (2) generating initial codes, (3) searching for themes, (4) reviewing themes, (5) defining themes, and (6) writing up (Joffe, 2012). The data set was coded by two coders independently following the recommendations of O’Connor and Joffe (2020). After familiarization with the data, the first author (AF) generated initial codes deductively based on recommendations for the design of ecological momentary interventions and relating to the main areas of interest of the study (i.e., facilitators of integration into everyday life, barriers to integration into everyday life, stress experiences, and music listening behavior), while additional codes were also generated inductively based on the data. During steps (3) and (4) of the analytical process, initial codes were then partly revised, refined, combined, or discarded, by returning to the dataset. This led to the generation of a coding frame and themes capturing common patterns across the dataset. A second coder (postgraduate student with a Bachelor’s degree in psychology) subsequently coded the dataset according to the coding frame. Krippendorff’s alpha was calculated as an indicator of intercoder reliability for each code individually, using the KALPHA macro for SPSS (Hayes and Krippendorff, 2007). It was defined *a priori* that codes with a Krippendorff’s alpha ≤0.75 would be reviewed for potential reasons of inconsistency among the coders and discussed until consensus was reached. The final intercoder reliabilities ranged from 0.79 to 1.00.

## Results

### Participant characteristics

**Figure 2:**
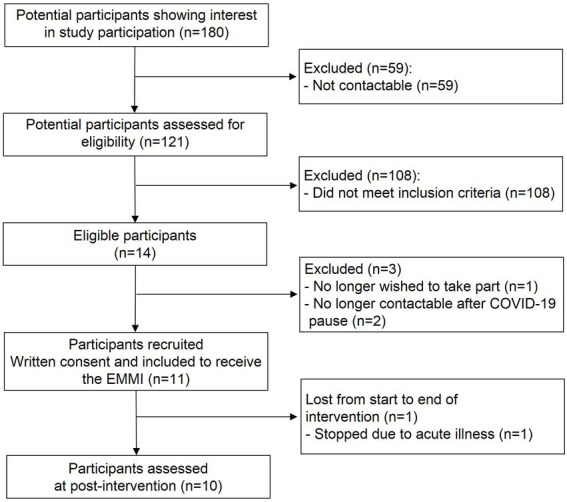
Study flowchart. EMMI, ecological momentary music intervention.

A study flowchart is shown in Figure 2. The final sample of this pilot study thus comprised *N* = 10 healthy female participants aged 23.5 ± 3.3 years (range 19–28 years) with a BMI of 20.0 ± 1.3 kg/m^2^ (range 18.2–22.5 kg/m^2^). Participants were highly educated: 2/10 participants had a Master’s degree, 4/10 had a Bachelor’s degree, and 4/10 had completed university entrance-level school-leaving examinations. Participants’ mean chronic stress levels based on the PSS lay at 20.4 ± 2.8 (range 15–24), with the majority (7/10) scoring 1 SD above the age- and sex-specific norm value (Klein et al., 2016). The mean BDI-II score of 5.9 ± 5.6 (range 0–17) indicated no clinically relevant depressive symptom severity in the present sample.

Half of the participants were tested between November 2019 and February 2020 and the other half between October 2020 and February 2021. Six participants received a study smartphone for data collection and four participants used their personal smartphones. With the exception of one participant, who brought her songs on a USB stick, all had access to a personal on-demand music streaming service. On average, participants had selected 19 ± 5 songs for their study playlist (range: 14–27).

### General compliance

The overall completion rate regarding the study protocol (i.e., automatically signaled and scheduled self-initiated data entries at awakening and bedtime – not including self-reported stressful experiences) across the whole study period was 69.5% ± 9.8% (530 missings/1,740 scheduled data entries). Completion rates varied across study periods and days, with the highest level of completion during the baseline period (77.9% ± 12.5%), followed by the post period (70.8% ± 12.7%), and the intervention period (67.7% ± 7.5%). The highest numbers of missings across individuals were found on the final day of each study period. Overall, response latencies for signaled data entries averaged 3:01 ± 9:02 min and response durations averaged 1:19 ± 2:05 min. Response latencies and durations in the intervention period were markedly shorter than those in the baseline and post period. Further details on compliance rates and response latencies/durations are provided in the Supplementary Table S2.

### Usage, compliance with, and perceived effects of the EMMI

#### Reported stressful experiences

Participants reported 77 stressful experiences across the whole study period. Twelve of these event-related data entries were reported in the baseline or post period (eight baseline, four post) and the remaining 65 were reported during the intervention period (i.e.; T_0_ data entry; see Figure 1; range: 3–11 per participant, 0–2 per day). Twenty-five of these latter entries were triggered following the semi-randomly scheduled prompts while the remaining 40 were self-initiated by the participants by pressing the button in the app.

We compared the 65 reports of stressful experiences with the retrospective reports provided at the bedtime data entry on the same intervention day. Twenty-four of the possible 180 bedtime data entries were missing, leaving 156 bedtime entries for comparison purposes. This descriptive analysis indicated that while 53 events were reported both during the day and at bedtime, 14 events were only reported retrospectively at bedtime. Based on the participants’ retrospective descriptions of the situations at the bedtime data entry, time pressure (e.g., hurrying to an appointment) and inappropriate circumstances to use the smartphone (e.g., driving the car, being in an important conversation, exam) seem to have been the main reasons for skipping reporting stressful events. In addition, 12 events were only reported directly during the day [either because participants missed the bedtime data entry (*n* = 7) or because they were unable to remember the number of events or remembered fewer events than reported during the day (*n* = 5)]. Therefore, on 85.2% of the study days, daytime and bedtime reports matched, while for the remaining days, there were either more stressful events reported at bedtime than during the day (54.2% of cases) or vice versa (45.8% of cases). Taking both daytime and bedtime reports into account, at least one stressful experience was reported on 39.5% of intervention days, while two or more stressful experiences were reported on 8.0% of intervention days.

For one of the 65 reported stressful experiences during the intervention period, further data were not provided, leaving 64 reports with additional situational information. For the majority of the experiences, participants had felt stressed for a duration of 5–20 min when starting the data entry (57.8%), followed by <5 min (20.3%), 21–45 min (12.5%), and >45 min (9.4%). “Work overload/time pressure” was the most frequently reported reason for initiating a data entry (40.6%), followed by “disagreement with another person” (17.2%), “external circumstances” (14.1%), “important appointment” (10.9%), “unexpected time delay” (6.3%), “other” (6.3%), and “problems of a person close to me” (4.7%). In addition, most of the stressful experiences were reported to be “happening at the moment” (54.7%) or were anticipated to “happen in the future” (31.3%), while only a minority were reported to have “happened in the past” (14.1%). For 40.6% of the reported experiences, participants indicated that they were currently perceiving additional stressful experiences apart from the one that led to the data entry.

For 4 out of the 65 stressful experiences reported during the intervention period, participants did not indicate whether or not they wanted to listen to music (i.e., incomplete data entry). On six occasions, participants decided not to listen to music. While they subsequently answered the T_1_ prompt accordingly, only on two of these six occasions did participants also provide data at the T_2_ prompt.

For 55 out of the 65 stressful experiences, participants followed the encouragement to listen to music. However, in three cases technical errors occurred and in one case the T_1_ prompt was not responded to, leaving 51 treatment occasions with valid self-report data at both T_0_ and T_1_. Finally, T_2_ prompts were not responded to on six of these 51 occasions, leaving a total of 45 treatment occasions with subjective data reported at all three measurement time points per stressful experience (T_0_, T_1_, T_2_).

The average response durations were 3:21 min ± 52 s (*n* = 61 entries) at T_0_, 2:18 min ± 2:32 min (*n* = 57 entries) at T_1_, and 1:50 min ± 1:22 min (*n* = 47 entries) at T2, with an additional 2 min for saliva sampling, respectively. The mean response latency for the prompt at T_1_ was 2:21 min ± 6:23 min. Finally, on average, 15:40 min ± 1:07 min passed between completing the response to the prompt at T_1_ and starting the response to the prompt at T_2_, indicating that when individuals responded to the T_2_ prompt, they answered it almost instantly.

#### Saliva sampling compliance and quantity

Saliva samples at T_0_ were provided for 59 of the 65 reported stressful experiences, with a mean saliva weight of 0.76 ± 0.49 g and three saliva weights below the recommended threshold of 0.2 g. Subsequently at T_1_, 52 saliva samples were taken promptly and could be matched to the corresponding data entries (four samples after no music listening, 48 samples after music listening), with a mean saliva weight of 0.81 ± 0.45 g and three saliva samples below the 0.2 g threshold. At T_2_, 46 saliva samples were taken adequately (two samples after no music listening, 44 after music listening), with a mean saliva weight of 0.73 ± 0.48 g and two samples weighing less than 0.2 g. Overall, participants provided complete data, that is subjective self-reports and saliva samples at all three measurement time points (T_0_, T_1_, T_2_), for 46/65 reported stressful experiences.

#### (Non-)music listening episodes

On the six occasions when participants decided not to listen to music, various reasons were indicated (e.g., “currently attending a course/lecture”, “being engaged in a conversation”, “hurrying to work”). For the remaining 51 stressful experiences, participants selected an average music listening duration of 12:53 min ± 7:10 min (range 4:39 min–39:12 min) according to their app-based data entries. This corresponds closely to the objectively tracked music listening data, with an elapsed mean duration of 9:00 min ± 7:00 min (range 0 min–38 min) from the start of the first to the start of the last song. Moreover, participants listened to an average of 3.5 ± 1.9 songs per stressful experience (range 1–10).

After music listening (at T_1_), participants reported that they liked the music very much (*M* = 4.6 ± 0.6 on a Likert scale from 1 to 5). Moreover, the music was perceived as rather calming (*M* = 35.9 ± 19.3, on a visual analogue scale from 0 to 100) and neither extremely sad nor happy (*M* = 51.1 ± 17.3, on a visual analogue scale from 0 to 100). On the majority of occasions (*n* = 31, 62%), participants would have liked to listen to music for longer than initially indicated. Loudspeakers (*n* = 33, 66%) were more frequently used than head−/earphones. In most instances, no other person was present while participants listened to music (*n* = 40, 80%).

#### Perceived immediate effects

**Figure 3:**
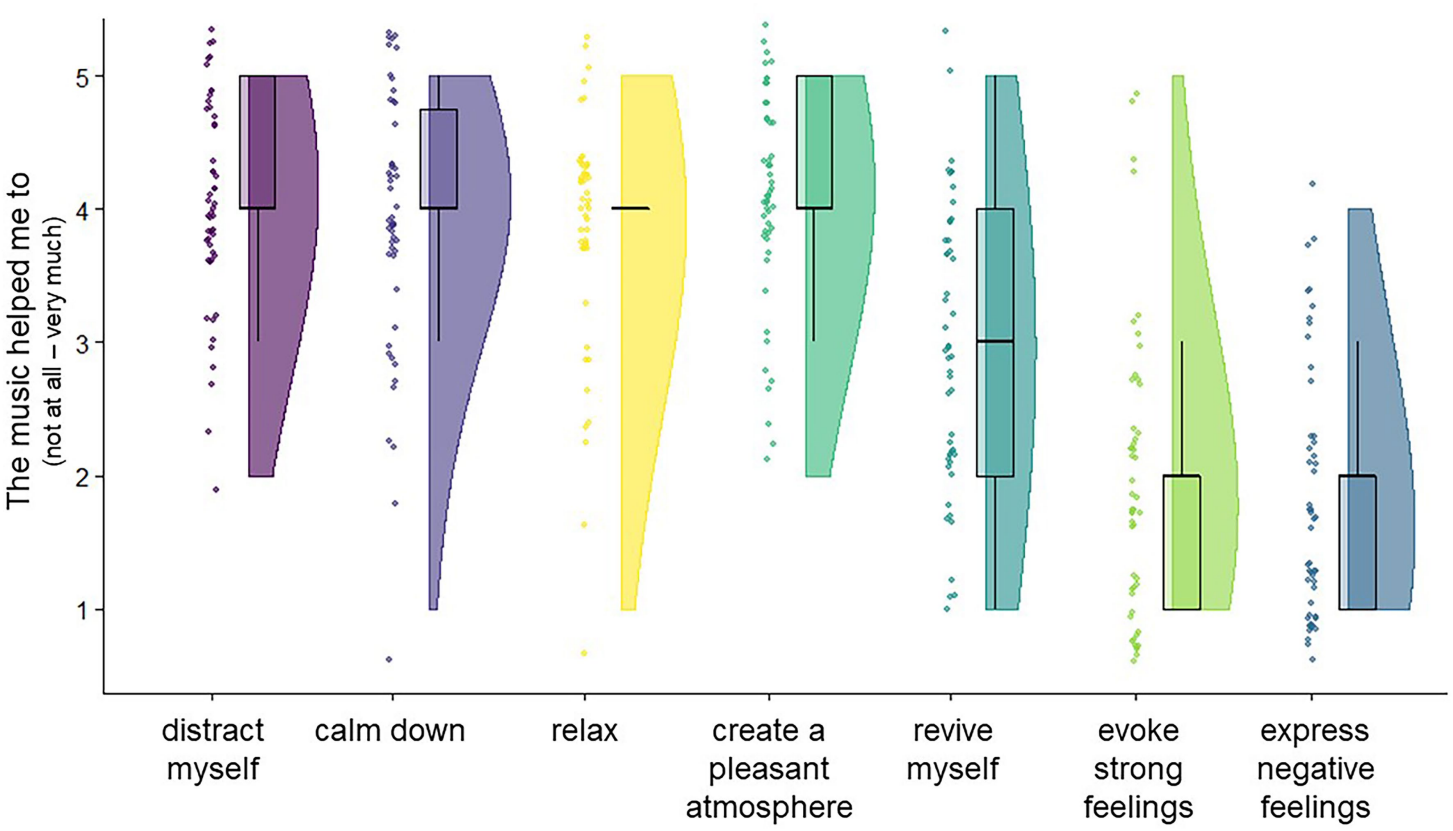
Perceived effects of music listening when following the encouragement to listen to music in moments of stressful experiences. Participants’ responses provided at T_1_ (after music listening) in moments of stressful experiences. Based on *n* = 50 events from *N* = 10 participants.

According to participants’ reports at T_1_, immediate benefits of music listening in moments of acute stressful experiences mainly encompassed being distracted (*M* = 4.1 ± 0.8) and creating a pleasant atmosphere (*M* = 4.1 ± 0.8), as well as calming down (*M* = 3.9 ± 0.9), and relaxing (*M* = 3.8 ± 0.8), see also Figure 3 for details.

### Usability and participant satisfaction

Based on participants’ responses regarding the usability and satisfaction questionnaire (see Table 2), it became apparent that the majority found the intervention usable, easy and intuitive to apply in daily life. Overall, satisfaction with the intervention seemed to be moderate to high, although there were also several lower scores. In this regard, participants’ statements during the PMI helped to identify specific facilitators and barriers to successfully integrating the study procedures into daily life and enabled a more nuanced understanding of participants’ (dis)satisfaction with the EMMI. Five major themes resulted from the analytic process: (1) *Methodological and technical determinants*, (2) *Situational circumstances matter*, (3) *Committed participant behaviors and attitudes*, (4) *Increased insight into individual stress fluctuations*, (5) *New and pleasant way of using music*. Details on codes, number of coded segments across interviews, number of participants discussing thematic content, as well as examples of participants’ statements are provided in the Supplementary Table S3.

The theme *Methodological and technical determinants* combines aspects of the study protocol and the functionality of the app that were perceived as either burdensome or helpful. These aspects were also related to participant satisfaction overall and provide crucial information indicating which components of the study design and the app should be maintained or require further adaptation in subsequent studies. Overall, many participants found the study participation cumbersome, mainly due to the frequent alarms throughout the day and the number of items that needed to be answered with each prompt. This was mostly evident during the baseline and post period, in which eight data entries, each including several items, were scheduled. Intervals between two successive prompts were sometimes perceived as too short, and prompts were perceived as bothersome when they interrupted daily activities (e.g., studying, watching TV). Furthermore, the fact that some alarms could not be postponed (e.g., +30 min after awakening, T_1_ and T_2_ in moments of stressful experiences) was mentioned as inconvenient. With regard to the intervention period, the need to provide three data entries, including saliva samples, for each reported stressful event was perceived as burdensome, and the number of items for each data entry was time-consuming. Furthermore, some participants reported technical difficulties, e.g., being disrupted by a semi-random prompt during music listening. On the positive side, participants reported that the app was very convenient and self-explanatory and that providing answers and saliva samples was straightforward. Moreover, the possibility to postpone the majority of scheduled data entries facilitated the integration of the study into daily life. Some participants found the intervention period overall to be easy to integrate into their daily life, as the semi-randomly scheduled prompts only included one single question and stressful experiences could be reported independently. Finally, two participants mentioned that the repeated prompts during the intervention period served as reminders to actually reflect upon their stress levels and to initiate a data entry once stressful situations were perceived.

The theme *Situational circumstances matter* describes contexts and circumstances that facilitate or impede the completion of data entries and following the encouragement to listen to music in moments of perceived stress. In this regard, participants reported that it was inconvenient to answer prompted data entries or initiate data entries if they were currently attending a course/lecture, working, or engaging with others (e.g., colleagues/clients). Additionally, being under time pressure (e.g., due to important appointments) made it difficult to listen to music or even start a data entry when feeling stressed. Nonetheless, participants named several circumstances in which music listening in moments of perceived stress was particularly easy to realize, including when being at home and when commuting (with head−/earphones).

The theme *Committed participant behaviors and attitudes* comprises active participant behaviors that helped them to integrate the study requirements into their daily schedules. Informing one’s social environment about study participation seemed to be one aspect in this regard. In most cases, friends and family showed an interest in the study, which might also have helped participants to stay engaged and facilitated saliva sampling and storage. Moreover, participants’ own interest in the study topic was evident during the interviews and may have enhanced their motivation to comply with the study protocol. Participants also mentioned that they explicitly planned ahead in order to be able to meet the study requirements (e.g., preparing saliva samples for the next morning the night before, identifying private places for saliva collection when in public). Furthermore, some participants mentioned that they needed one or two days to familiarize themselves with the study procedures during the baseline period and read the study manual at the beginning, which helped them to accommodate to the study protocol.

The theme *Increased insight into individual stress fluctuations* describes a major advantage that participants reported as a result of their study participation, namely an increased reflection on and awareness of their own stress levels and how these fluctuated throughout the day, due to the repeated prompts. Some also identified daily stressors that repeatedly elicited feelings of stress, which helped them to cope better. Moreover, several participants mentioned that their understanding of “feeling stressed’ had changed, for instance due to an increased awareness that even small things in daily life can qualify as stressors and that stress can be elicited by more than just time pressure (e.g., social conflicts, lack of control).

The final theme, *New and pleasant way of using music*, concerns participants’ experiences when engaging in music listening in moments of acute stress. The theme also includes differences to regular music listening behavior, as well as participants’ satisfaction with their selected playlist and playlist characteristics. Many participants stated that prior to their participation in the study, music listening had at times helped them to relax, but they had not listened to music when feeling acutely stressed. Instead, music listening was rather a background activity when engaging in other tasks or was used for the purpose of activation rather than calming down. However, due to the encouragements by the app in moments of perceived stress, participants were reminded to take a break from their current activity and to listen to music in a deliberate manner, which were obviously two important intervention components that were evaluated as supportive. Characteristics of the playlist that were mentioned as particularly beneficial were that the songs were perceived as relaxing (although some participants mentioned that it was not “relaxing” music *per se*), and that they had chosen songs that they liked a lot and with which they were familiar. Some participants also mentioned that they had chosen songs associated with positive memories (e.g., spending time with friends). Further positive aspects were that participants had not heard the songs for a while before study participation and having a large selection of music from which to choose. All participants except for one stated that they liked their playlist a lot and that the selected songs were appropriate for use in moments of acute stress. One participant stated that she would have preferred to listen to different songs than those on her playlist. Interestingly, she stated that the songs she had selected were too calming and somewhat sadder than those she usually listens to, and that when feeling stressed, she typically also felt tired and would have preferred to listen to more activating music, in order to be revived and gain new energy. Finally, although not asked directly, several participants stated that they had experienced a new way of consuming music that had helped them to cope with momentary stressful experiences and that they might continue to try this out in the future.

## Discussion

### Summary of principal findings

We aimed to test the feasibility of a newly developed EMI delivering music in moments of acute stressful experiences in daily life. Taken together, the findings of this uncontrolled pilot study indicate the feasibility of the EMMI. On most of the study days, the number of reported stressful experiences during the day corresponded to the number of retrospectively reported events at bedtime. In addition, the overall number of reported stressful experiences in this study was comparable to previous daily diary studies [e.g., 39.5% of days with at least one stressor in the present study; 39.4% in the study by Almeida et al. (2002), and 36% in the study by Bellingtier et al. (2017)]. Furthermore, for the majority of reported stressful experiences, participants were able to engage in music listening for a reasonable duration and reported beneficial immediate effects such as calmness, relaxation, and distraction. The findings also suggest that stressful situations in which music listening was not possible often required the participants to engage in a conflicting activity (e.g., interacting with clients at work; hurrying to an appointment). Consequently, an immediate active behavior, rather than an emotion-focused behavior like music listening, was perceived as necessary and adequate to manage the situation. In addition, participants’ overall satisfaction with the usage of the EMMI *per se* was high.

Nonetheless, certain subcomponents of the study protocol, such as the number of prompts during the baseline and post period, were perceived as burdensome. Participants also found it laborious to provide three data entries, including saliva samples, for each stressful experience, which was associated with a rather long response duration according to the app’s log data. This might have impeded the reporting of some stressful experiences and/or compliance rates, as indicated by the fact that on only 46 out of the 65 reported events were both self-reports and saliva samples provided at all three measurement time points (T_0_, T_1_, T_2_).

Future larger-scale studies might benefit from several specific adaptations to the general study protocol, and it should be considered that participants will not be able to act on the encouragement to listen to music in 100% of acute stressful experiences in daily life. Overall, however, our findings suggest that the EMMI can be successfully implemented.

### Recommendations for future research

The present feasibility study helped to identify several aspects of the study design that we would recommend to consider for future just-in-time ecological momentary (music) intervention studies targeting acute stressful experiences. A summary of the key recommendations is displayed in Table 3.

**Table 3:** Key recommendations for the design of just-in-time EM(M)Is for the reduction of acute stress.

1	Communicate a clear-cut and theory-driven definition of “stress” to participants at the beginning of the study in order to reach a shared understanding of the applied stress-related measures
2	Plan the number of days, prompts per day, and overall study duration carefully. Keep participant burden as low as possible
3	Allow participants to familiarize with the study protocol at home (e.g., provide a study manual, include exercise days)
4	Keep response durations in moments of stressful experiences as short as possible (e.g., choose few items, let participants answer while taking saliva samples)
5	Participants’ self-compiled study playlists should contain music pieces that they like a lot, perceive as relaxing, and like to listen to when feeling stressed

General recommendations on EMA/I protocol designs can be found in Shiffman et al. (2008), Mehl and Conner (2013), Steinhart et al. (2019), Balaskas et al. (2021). EMA, ecological momentary assessment; EMI, ecological momentary intervention; EMMI, ecological momentary music intervention.

First, in the present study, we thoroughly instructed participants about the precise circumstances in which they should initiate a data entry, that is which characteristics a situation should meet to qualify as a “stressful experience,” based on the transactional stress theory (Lazarus and Folkman, 1984). Researchers have not yet agreed upon a single definition of “stress,” and laypersons’ definitions are even more diverse (Kinman and Jones, 2005). Therefore, we recommend that in future EMIs targeting stress-related feelings and/or appraisals in daily life, a clear-cut and theory-driven definition of stress should be communicated between researchers and participants. According to the theme *Increased insight into individual stress fluctuations* we also found that the participants’ understanding of “feeling stressed” had changed and that they gained more awareness on their stress fluctuations and stress-eliciting situations. Certainly, different theoretical conceptualizations of the stress phenomenon can be applied depending on the context of the study and the research questions. Nevertheless, we argue that to be able to adequately interpret study findings, it is important to make participants aware of the exact meaning of the applied stress-related measures from the researchers’ perspective (for in-depth discussions on the measurement of stress see Harkness and Monroe, 2016; Epel et al., 2018).

Moreover, if a baseline and post period are of interest, we suggest that fewer than eight data entries per day should be implemented if possible. Although it is not uncommon for ambulatory assessment studies to successfully implement up to 14 prompts or more per day (e.g., Greb et al., 2019), considering an already long overall data collection period (i.e., 24 days in the present study), a large number of prompts per day may have negatively influenced participants’ general compliance. The quantitative results as well as participants’ perceptions summarized in the theme *Methodological and technical determinants* indicate that compliance with the study could be improved in a future study by adapting the number of study days and prompts per day. For instance, it could be advantageous to include more days in the baseline and post period, with fewer data entries per day. This would support the familiarization with the study procedures during the first days of the study and would also compensate for the lower number of prompts per day. Importantly, though, the number prompts per day and the number of days should ultimately be primarily decided based on the research questions and phenomena of interest (Shiffman et al., 2008).

Unsurprisingly, the intervention period was perceived as easier to integrate into daily life than the baseline and post periods according to the PMIs (theme *Methodological and technical determinants*). This can be attributed to the possibility to quickly answer the scheduled prompts (that included only one single item) combined with the possibility to independently initiate data entries in moments of stressful experiences. Nonetheless, there was a relatively high number of missing data regarding the semi-randomly scheduled one-item prompt. This could indicate that the repeated single-item questions might have been perceived as unnecessary if participants were not feeling stressed, and/or may reflect a lower motivation to answer the item during a prolonged period of data collection (here: 18 days) in daily life. Nonetheless, some participants reported that the repeated prompts reminded them to actually reflect upon, and increased their awareness of, momentary stress levels and may therefore also have facilitated the self-reporting of stressful experiences. In addition, around 30% of the recorded stressful experiences were gathered due to the scheduled prompts, underlining their general usefulness in the present study. One way to further improve the prompting schedule in future studies might be to agree on a certain number of reminder prompts with each participant during the first introductory session and to schedule more prompts in periods when participants might anticipate more stressful experiences (Schmiedek and Neubauer, 2020). In this way, the study design would be tailored even more to the individual’s needs, and the opportunities to report stressful experiences might increase. Moreover, a higher number of prompts might be generally helpful in the first days of the intervention period, while fewer might be needed later on, assuming that individuals become more aware of their individual stress perceptions throughout study participation, as reported during the PMIs.

Importantly, although perceived as burdensome (theme *Methodological and technical determinants*), we consider three data entries per stress report necessary to be able to capture immediate effects from before to after (non-)music listening, as well as to assess time-delayed alterations in cortisol levels due to the temporal response dynamics of the HPA axis (Schlotz et al., 2008). In this regard, our findings emphasize the need to carefully adjust necessary response durations for each measurement point in the context of stressful experiences. For example, participants took around 3 min to complete the initial data entry (= T_0_) and an additional 2 min to collect a saliva sample. While the latter duration should remain unchanged, as it enabled the collection of sufficient saliva for biochemical analyses of salivary biomarkers, anticipating an overall response duration of ~5 min could have prevented participants from even starting a data entry when under time pressure. We suggest two possible solutions for future studies: first, reducing the number of items to an absolute minimum, and second, implementing the possibility to collect saliva during (and not only after) item responding. This would substantially reduce the overall response times as participants would already be accumulating saliva while answering the questions. Crucially, a countdown should be implemented to signal that the 2 min for saliva accumulation have elapsed and participants should start transferring saliva into the tube.

Another important EMI design aspect that is worth considering is the “threshold” of perceived stress that triggers treatment delivery. In the present study, we did not pre-specify a particular level of momentary stress that needed to be reached before the app encouraged participants to listen to music. Rather, after providing participants with a definition of a “stressful experience,” we relied on their own momentary evaluations. Previous studies have chosen different approaches to “just-in-time” treatment delivery. For instance, Neal-Barnett et al. (2019) predefined a score that needed to be reached before the treatment (music listening) was offered, whereas in their proof-of-concept study, Smyth and Heron (2016) based the delivery of treatment (written messages reminding participants to apply stress management skills) on “high” levels of stress, defined as being ≥1 SD above the person-centered mean of each participant individually. While it currently seems to be too early to give a general recommendation on a “best-suited” approach, it would certainly be interesting in future studies to combine our approach with a data-driven approach, e.g., delivering the treatment when a certain score (e.g., person-centered mean stress level + 1 SD, with mean levels derived from the baseline period) is exceeded while additionally providing the possibility to self-initiate data entries in moments of (self-perceived) need.

Concerning our treatment (i.e., listening to a self-selected music playlist), the theme *New and pleasant way of using music* resulting from the PMI revealed that one of our 10 participants would have liked to change the songs selected for her playlist, as she did not find them to be appropriate when perceiving stress. We instructed participants to select songs that they liked very much and perceived as calming/relaxing. Although the majority of our participants indicated that their playlists were well chosen and that the music had helped them to calm down, it might be advisable to slightly adapt the instructions in future studies by clarifying that participants should select songs they would like to listen to *when feeling stressed* (Groarke et al., 2019). Moreover, participants needed to decide *a priori* on the duration of music listening in moments of stressful experiences (between 5 and 30 min). Although this should provide the possibility of adjusting the treatment to the participants’ momentary situation, participants might have been prompted by the app to stop music listening in an inopportune moment (e.g., in the middle of a highly liked song). This is supported by the finding that participants would have liked to listen longer to the music than initially indicated on the majority of occasions. One possible solution could be to allow participants to stop music listening at any time that suited them between 5 and 30 min and to implement a technically more versatile system that registers when individuals stop music listening on the smartphone and then activates the EMA app with the subsequent questions.

Finally, the present study demonstrates that listening to music as a treatment is not practical across all situations perceived as stressful, that is a 100% adherence to the treatment is not to be expected (as is probably the case with many EMIs). Thus, the findings of the present study are crucial, as they hint at a to-be-expected number of reported stressful experiences and music listening episodes in relation to such events. Such information is valuable with respect to the design and power considerations of future larger-scale RCTs aiming to identify a causal relationship between music listening in moments of stressful experiences and psychobiological stress levels in daily life. In this regard, Schmiedek and Neubauer (2020) suggest a “within-person encouragement design,” which combines experimental within-person manipulations and the use of random encouragements as instrumental variables when strict treatment adherence is not plausible. In the present context, this implies that participants could be randomly encouraged to listen to music for a subset of stressful experiences and not encouraged for another subset, using a predefined ratio (e.g., 50:50). If the assumption holds that any effect of the encouragement to engage in music listening on psychobiological stress levels is fully mediated by the treatment itself (i.e., music listening) and provided that individuals are more likely to engage in music listening due to the encouragement, implementing such an intraindividual manipulation would allow future studies to address temporal within-person causal processes.

### Strengths and limitations

To the best of our knowledge, the present study is the first to describe an intervention approach targeting daily stress responses that arise as individuals go about their everyday life. Furthermore, to assess feasibility from various perspectives, we used a combination of quantitative and qualitative data: app log data and participants’ EMA ratings provided information on in-the-moment compliance, usage, and perceived effects, while (non-EMA) questionnaire and semi-structured interview data yielded a valuable understanding of facilitators and barriers to the integration of the EMMI into daily life. Due to this mixed methods approach, we gained important insights into the feasibility of the EMMI at an early stage of the intervention development process and were able to derive specific implications for the design of future just-in-time interventions targeting stressful experiences.

Nonetheless, several limitations of the present study need to be critically considered. First, our sample of *N* = 10 was small. However, the main aim of the study was to acquire knowledge on the feasibility and functionality of the EMMI regarding technical and content-based aspects of the study protocol before implementing the EMMI in larger samples (O’Cathain et al., 2019). Moreover, half of the study participants were tested before and the other half after the outbreak of the COVID-19 pandemic. Thus, we cannot rule out that the reporting of stressful experiences was affected by stay-at-home and physical distancing orders. Indeed, participants taking part before the pandemic reported more stressful experiences compared to individuals taking part during the pandemic, while they did not differ regarding demographic variables, depressive symptoms, chronic stress, general compliance rate, perceived usability/satisfaction, and duration of music listening in moments of stressful experiences (data not shown). This is unfortune on the one hand, as it indicates that the pandemic-related restrictions were associated with a lower number of minor stressors of daily life registered in this study. On the other hand, our results show that the EMMI is applicable in daily life even during a global crisis. A further limitation concerns a potential self-selection bias of our sample. Participants were aware that the study was about listening to music in moments of perceived stress. Consequently, it can be assumed that individuals interested in participating generally liked listening to music in daily life and our findings are therefore not representative of, and cannot be generalized to, individuals who do not like music (e.g., individuals with music anhedonia). Finally, as we only recruited women for the present feasibility study, our findings might not translate to men or non-binary individuals. Future studies, particularly those investigating effectiveness, should consider sex/gender-specific effects of the EMMI.

### Conclusion and outlook

Music has a long tradition as a means of stress reduction and is increasingly being implemented in health care settings (Collins and Fleming, 2017). Moreover, music listening is an activity of everyday life that is highly liked across different cultures and age groups. We propose the application of music listening in moments of acute stress responses that occur as individuals go about their everyday life when confronted with daily stressors. The present study confirmed the feasibility of our newly developed EMMI and provided valuable implications for the design of subsequent studies. Besides mere self-reports, our study protocol includes objective measures of music listening as well as biological stress markers. This multidimensional measurement approach will enable a comprehensive and fine-grained analysis of music-induced effects on the human stress response in the natural environment. As a next step in this direction, the present findings can be used to inform larger-scale studies (e.g., RCTs) that focus on the effectiveness of the EMMI. On a wider scope, future developments in the innovative field of mobile health might promote the implementation of music listening treatments that are even more tailored to an individual’s preferences and momentary needs when experiencing stress, for instance in terms of adjusted music characteristics and music listening duration.

## Data availability statement

The raw data supporting the conclusions of this article will be made available by the authors, without undue reservation.

## Ethics statement

The studies involving human participants were reviewed and approved by Ethics Review Board of the University of Vienna, Vienna, Austria. The patients/participants provided their written informed consent to participate in this study.

## Author contributions

AF and UN conceived the study and were in charge of overall direction, coordination, and planning. AF collected and analyzed the data and wrote the first draft of the manuscript. All authors contributed to the article and approved the submitted version.

## Funding

This work was supported by the University Research Platform “The Stress of Life (SOLE) – Processes and Mechanisms underlying Everyday Life Stress.”

## Conflict of interest

The authors declare that the research was conducted in the absence of any commercial or financial relationships that could be construed as a potential conflict of interest.

## Publisher’s note

All claims expressed in this article are solely those of the authors and do not necessarily represent those of their affiliated organizations, or those of the publisher, the editors and the reviewers. Any product that may be evaluated in this article, or claim that may be made by its manufacturer, is not guaranteed or endorsed by the publisher.
