# Supplementary material for: An ecological momentary music intervention for the reduction of acute stress in daily life: A mixed methods feasibility study
Source: Front Psychol. 2022 Sep 29;13:927705. doi: 10.3389/fpsyg.2022.927705 (PMC9558284; doi:10.3389/fpsyg.2022.927705)
Supplement: Supplementary file 1 [file Data_Sheet_1.docx]

Supplementary Material

An ecological momentary music intervention for the reduction of acute stress in daily life: a mixed-methods feasibility study; Anja C. Feneberg, Urs M. Nater

| **Table S1**. EMA Items used in this study. | | |
| --- | --- | --- |
| **Assessment time point** | **German** | **English translation** |
| **T_0_** | Wie lange fühlen Sie sich bereits gestresst?   - weniger als 5 Minuten - 5-20 Minuten - 21-45 Minuten - > 45 Minuten | How long do you already feel stressed?   - less than 5 minutes - 5-20 minutes - 21-45 minutes - > 45 minutes |
|  | Bitte wählen Sie die Kategorie aus, die am ehesten beschreibt, durch welche Begebenheit Ihr momentanes Stresserleben ausgelöst wurde:   - Unstimmigkeiten mit anderer/n Person/en - Unerwartete Verzögerungen im geplanten Zeitablauf - Zu viele Aufgaben und/oder zu wenig Zeit zur Erledigung/Zeitdruck - Wichtiger Termin - Umgebungsbedingungen - Diskriminierung - Probleme einer nahestehenden Person - Anderes | Please select the category that most closely describes the occurrence eliciting your momentary stress experience:   - Disagreement with another person - Unexpected time delay - Work overload and/or too less time to complete work/time pressure - Important appointment - External circumstances - Discrimination - Problems of a person close to me - Other |
|  | Die stressauslösende Situation bzw. das stressauslösende Ereignis...   - liegt in der Vergangenheit - findet aktuell statt - steht noch bevor | The stress-eliciting situation/the stress-eliciting occurrence…   - happened in the past - happens at the moment - will happen in the future |
|  | Gibt es noch weitere Begebenheiten, durch die Ihr momentanes Stresserelben zusätzlich ausgelöst wurde? (bitte alle zutreffenden markieren)   - Unstimmigkeiten mit anderer/n Person/en - Unerwartete Verzögerungen im geplanten Zeitablauf - Zu viele Aufgaben/Verpflichtungen und/oder zu wenig Zeit zur Erledigung/Zeitdruck - Wichtiger Termin - Umgebungsbedingungen - Diskriminierung - Probleme einer nahestehenden Person - Anderes | Are there any further events that additionally elicited your momentary stress experience? (please tick all that appy)   - Disagreement with another person - Unexpected time delay - Work overload and/or too less time to complete work/time pressure - Important appointment - External circumstances - Discrimination - Problems of a person close to me - Other |
| **T_1_**  ***after music listening*** | Hat Ihnen die gehörte Musik gefallen?  überhaupt nicht (1) – sehr (5) [Likert-Skala] | Did you like the music you listened to?  not at all (1) – very (5) [Likert scale] |
|  | Die Musik, die Sie gehört haben, war…  traurig (0) – fröhlich (100)  [Visuella Analogskala] | The music you listened to, was …  sad (0) – happy (100)  [Visual analog scale] |
|  | Die Musik, die Sie gehört haben, war…  beruhigend (0) – energetisierend (100)  [Visuella Analogskala] | The music you listened to, was …  calming (0) – energizing (100)  [Visual analog scale] |
|  | Hätten Sie gern noch länger Musik gehört?   - Ja - nein | Would you have preferred to continue music listening?   - Yes - No |
|  | Haben Sie über Kopfhörer oder Lautsprecher Musik gehört?   - Kopfhörer - Lautsprecher | Did you listen to music via head-/earphones or loudspeakers?   - Head-/earphones - Lourdspeakers |
|  | Welche Person(en) waren anwesend, während Sie Musik gehört haben?   - ich war allein - Freunde - Bekannte - Familie - Partner - Unbekannte | Who was present while you listened to music?   - I was alone - Friends - Acquaintances - Family - Partner - Unknown persons |
|  | Die Musik hat mir geholfen,...^a^   - mich abzulenkten - mich zu beruhigen - negative Gefühle auszudrücken - neue Kraft zu tanken - eine angenehme Atmosphäre zu schaffen - starke Gefühle hervorzurufen - mich zu entspannen   überhaupt nicht (1) – sehr (5) [Likert-Skala] | The music helped me to…^a^   - distract myself - calm down - express negative feelings - revive myself - create a pleasant atmosphere - evoke strong feelings - relax   not at all (1) – very (5) [Likert scale] |
| **Bedtime** | Haben Sie heute Momente erlebt, in denen Sie sich gestresst fühlten?   - ***Ja*** - Nein | Did you experience stressful moments today?   - ***Yes*** - No |
|  | *Bei „****Ja****“*: Wieviele stressreiche Momente haben Sie heute insgesamt erlebt?   - 1 - 2 - 3 - >3 - Ich weiß es nicht | *If “****Yes****”*: How many stressful moments did you experience today in sum?   - 1 - 2 - 3 - >3 - I don’t know |
|  | *Bei „****Ja***“: Haben Sie die stressreichen Situationen in der App aufgezeichnet?   - Ja - ***Nein*** - ***Ich weiß es nicht*** | *If “****Yes****”*: Did you record the stressful moments in the app?   - Yes - ***No*** - ***I don’t know*** |
|  | *Bei „****Nein****“/“****Ich weiß es nicht****“:* Können Sie diese Situation/en (einzeln) kurz beschreiben? [Freitexteingabe] | *If “****No****”/”****I don’t know****”*: Could you describe these situation(s) briefly?  [Open text format] |

*Notes*. Displayed are items relevant for the present feasibility study. T_0_=initiated data entry in moments of stressful experiences, T_1_=signaled data entry after treatment (music listening). EMA, Ecological momentary assessment. ^a^Items adapted from the Brief-Music in Mood Questionnaire (Saarikallio, 2012).

| **Table S2**. Compliance rates, response latencies, and response durations for scheduled data entries separated by study period. | | | | | | |
| --- | --- | --- | --- | --- | --- | --- |
|  | Baseline | | Intervention | | Post | |
|  | M (SD) | Min; Max | M (SD) | Min; Max | M (SD) | Min; Max |
| Missings (n)^ab^ | 5.3 (3.0) | 2; 12 | 40.7 (9.4) | 15; 52 | 7.0 (3.0) | 3;13 |
| Completion rate (%)^b^ | 77.9 (12.5) | 50.0; 92.0 | 67.7 (7.5) | 59.0; 88.0 | 70.8 (12.7) | 46.0; 88.0 |
| Response latency^c^ | 4 min 45 sec (9 min 16 sec) | 3 sec;  37 min 29 sec | 1 min 56 sec (6 min 53 sec) | 1 sec;  1 h 13 sec | 8 min 44 sec (16 min 28 sec) | 3 sec;  59 min 56 sec |
| Response duration^d^ | 2 min 47 sec (1 min 49 sec) | 39 sec;  13 min 55 sec | 12 sec  (26 sec) | 1 sec;  5 min 17 sec | 2 min 01 sec  (1 min 10 sec) | 38 sec;  8 min 47 sec |

^a^Maximal number of scheduled data entries: baseline = 240, intervention = 1260, post = 240.

^b^Data averaged on the participant level.

^c^Data averaged across semi-randomly signaled data entries.

^d^Data averaged across semi-randomly signaled data entries, morning, and bedtime data entries (excluding time for saliva sample collection).

| **Table S3**. Themes, Codes, and Quotes from post-monitoring interviews. | | | | |
| --- | --- | --- | --- | --- |
| **Themes and codes** | **segments n** | **participants n** | **K**  **alpha^1^** | **Exemplary quotes** |
| ***Methodological and technical determinants*** | | |  |  |
| *Difficulties* | | | | |
| Amount of prompts, questions per prompt | 9 | 4 | 0.88 | *- I think the initial phase and the final period were rather… hmm.. because it was a little often.*  *- I am actually stressed when I have to do something or when I’m under time pressure and if I had to fill out the questionnaire then, it just took so much time.* |
| Three data entries/saliva samples per stress event | 4 | 3 | 1.00 | *Those three times, that was actually quite a lot of effort* |
| Short intervals between prompts | 4 | 3 | 1.00 | *And sometimes I thought it was hard, or rather several times, for example during the baseline or post period, when I am asked again an hour later somehow, then it hasn’t really changed for me in the last hour.* |
| Signals disrupting current activities/daily routine | 14 | 6 | 0.83 | *Especially when I was watching a movie or something, right in the middle of it, that was a little annoying. But yeah, it was bearable. Well, it was a little annoying.* |
| Mistyping | 6 | 3 | 1.00 | *… with the app.. yeah two times it happened to me, that I clicked on accept the alarm instead of postpone.* |
| Technical difficulties | 12 | 6 | 0.96 | *During the intervention period, everything was also okay. Only sometimes, when I wanted to initiate the bedtime data entry, it didn’t work and then I needed to restart the smartphone.* |
| *Facilitators* |  |  |  |  |
| Handy app and self-explanatory procedures | 11 | 8 | 1.00 | *Yeah, handling the app I thought was very easy and the saliva samples were always well labeled.* |
| Intervention period easy to integrate into daily routine | 11 | 7 | 1.00 | *…especially in the intervention phase it was easy to start and it didn’t significantly influence my everyday life in some way* |
| Prompts in intervention period serve as reminders to self-initiate ‘stress’ reporting | 2 | 2 | 1.00 | *And when for example I experienced these moments of stress, then I very often automatically responded when I was asked: Are you stressed? I said no. And then I thought: But yes I am, and then I started a separate stress measurement.* |
| Possibility to postpone data entries | 3 | 3 | 1.00 | *I sometimes postponed them, the alarms. I postponed them for a few minutes, if it wasn’t the right time then.* |
| ***Situaitonal circumstances matter*** |  |  |  |  |
| Inconvenient circumstances to provide data entries (general) | 14 | 6 | 0.79 | *…especially when you’re at work or at university or something, well, I think it’s a bit difficult.* |
| Inconvenient circumstances to engage in music listening (intervention period) | 24 | 9 | 1.00 | *…a lot of the times it was like that, I have been at work when I was stressed and then it just didn’t work, I just couldn’t. I just couldn’t listen to music then.* |
| Convenient circumstances to engage in music listening | 12 | 7 | 0.95 | *- Most of the times I listened to music alone. Or actually always.*  *- …other than that the thing concerning music listening, I actually only always did it if I was at home.*  *- But yeah, if you take the time for something like that or when you’re on the road, it usually works out.* |
| ***Committed participant behaviors and attitutes*** | |  |  |  |
| Engaging social environment | 12 | 9 | 1.00 | *Sure, if the alarm is ringing somehow, then people would ask, or if I run around with two smartphones, people would also ask why I am running around with two smartphones. And actually all of them were very interested in the study, they all thought it was really interesting.* |
| Personal interest in study topic | 9 | 6 | 0.91 | *Music is just very important to me. Therefore, music, a very interesting topic to me, I’d like to do some research, too.* |
| Planning ahead | 16 | 6 | 0.91 | *Those samples, I always put them there in the morning, I mean in the evening, so that I won’t forget about it in the morning.* |
| Getting familiar with the study procedures | 7 | 3 | 0.83 | *In the beginning it was a bit … reading into it, with the measurements, at what time you’re reminded to do them and at what time I should start, well, the measurement myself. But it was only a little bit, having to read into it, that you get familiar with it. But after that, especially after the first two days, it was pretty easy to handle.* |
| Private places for collection of saliva samples | 6 | 5 | 0.91 | *And at work also not right in front of the customer, but rather at the toilet, if it was somehow possible.* |
| ***Increased insight into individual stress fluctuations*** | | |  |  |
| Increased reflection on and awareness of stress fluctuations and stress elicitators | 22 | 9 | 1.00 | *- I quite liked the questions, because by being asked so often about how I felt, I started to reflect a little more and then actually… That always depends on the situation and I quite like that, that you’re not only asked about it once a day but several times, because it does actually change. - So, I learned a few things…that I often put stress on myself even though I didn’t have to. That’s what I realized. And also that I’m always stressed by the same topic.* |
| Improved understanding of what ‘feeling stressed’ means | 5 | 5 | 0.89 | *- That I think to myself, that even minor things matter, that these are also stressful.. It doesn’t have to be, I mean I don’t have to go through anything really bad to experience some kind of stress. - Before, I mostly only thought of stress as time pressure.* |
| ***New and pleasant way of using music*** |  |  |  |  |
| New way of listening to music (e.g., conscious listening, different music characteristics than usual, in acute stress moments) | 37 | 10 | 0.90 | *However, I did think that listening to music was kind of pleasant, even if I didn’t imagine it that way before. Because, I need to say, when I then just simply took the 5 to 10 minutes time, that it was actually quite pleasant.* |
| Playlist satisfaction   - satisfied - not satisfied | 9  1 | 9  1 | 0.95 | *- I* *really liked the music that I selected for the playlist.*  - *Yes, sometimes I would have rather liked to listen to a different kind of music.* |
| Playlist characteristcs | 26 | 10 | 0.93 | *Because I chose music I didn’t really listen to the week before, so that’s why it was pretty cool, because I haven’t listened to it in a while, but they weren’t really the calmest songs; of course it was songs I also relax to, but not classical music or something, but also songs I usually also listen to, not just for relaxation.* |
| New stress management strategy after study participation | 8 | 5 | 0.94 | *I can imagine that I will do that in the future, too, like creating a playlist like that and listening to it when I’m stressed out.* |

*Notes*. Displayed are themes and codes, number of segments per code, and number of participants contributing to the respective code; and quotes based on post-monitoring interviews from N=10 participants. ^1^Krippendorff’s alpha between coder 1 (AF) and coder 2 (psychology student with a Bachelor’s degree).

**References**

Saarikallio S. Development and Validation of the Brief Music in Mood Regulation Scale (B-MMR). *Music Perception: An Interdisciplinary Journal* 2012; 30: 97–105.
